# Supplementary material for: Flexible and scalable diagnostic filtering of genomic variants using G2P with Ensembl VEP
Source: Nat Commun. 2019 May 30;10:2373. doi: 10.1038/s41467-019-10016-3 (PMC6542828; doi:10.1038/s41467-019-10016-3)
Supplement: Supplementary file 1 — Supplementary Information [file 41467_2019_10016_MOESM1_ESM.pdf]

Flexible and Scalable Diagnostic Filtering of Genomic Variants using G2P with Ensembl VEP

Thormann et al.

Supplementary Tables

Supplementary Table 1:G2P Gene Categories

Category	Description
**Confirmed RD Gene**	Plausible disease-causing mutations* within, affecting or encompassing an interpretable functional region** of a single gene identified in multiple (>3) unrelated cases/families with a relevant disorder***
**Confirmed RD Gene**	Plausible disease-causing mutations within, affecting or encompassing cis-regulatory elements convincingly affecting the expression of a single gene identified in multiple (>3) unrelated cases/families with a relevant disorder
**Confirmed RD Gene**	As definition 1 and 2 of Probable RD Gene (see below) with aRDition of convincing bioinformatic or functional evidence of causation e.g. known inborn error of metabolism with mutation in orthologous gene which is known to have the relevant deficient enzymatic activity in other species; existence of animal mode which recapitulates the human phenotype
**Probable RD Gene**	Plausible disease-causing mutations within, affecting or encompassing an interpretable functional region of a single gene identified in more than one (2 or 3) unrelated cases/families or segregation within multiple individuals within a single large family with a relevant disorder
**Probable RD Gene**	Plausible disease-causing mutations within, affecting or encompassing cis-regulatory elements convincingly affecting the expression of a single gene identified in in more than one (2 or 3) unrelated cases/families with a relevant disorder
**Probable RD Gene**	As definitions of Possible RD Gene (see below) with convincing bioinformatic or functional evidence of causation e.g. known inborn error of metabolism with mutation in orthologous gene which is known to have the relevant deficient enzymatic activity in other species; existence of animal mode which recapitulates the human phenotype
**Possible RD Gene**	Plausible disease-causing mutations within, affecting or encompassing an interpretable functional region of a single gene identified in one case or segregation within multiple individuals within a small family with a relevant disorder
**Possible RD Gene**	Plausible disease-causing mutations within, affecting or encompassing cis-regulatory elements convincingly affecting the expression of a single gene identified in one case/family with a relevant disorder
**Possible RD Gene**	Possible disease-causing mutations within, affecting or encompassing an interpretable functional region of a single gene identified in more than one unrelated cases/families or segregation within multiple individuals within a single large family with a relevant disorder
**Both RD and IF**	Plausible disease-causing mutations within, affecting or encompassing the coding region of a single gene identified in multiple (>3) unrelated cases/families with both a relevant disorder and an incidental (non-developmental) disorder

* Plausible disease-causing mutations = Recurrent de novo mutations convincingly affecting gene function OR Rare, fully-penetrant mutations - relevant genotype never seen in contols; ** Interpretable functional region = e.g. ORF in protein coding genes OR miRNA stem or loop; *** relevant disorder = Disorder or clinical presentation for which the diagnostic filtering is designed to diagnose

Supplementary Table 2: Allelic requirement categories

Category	Description
**Monoalellic**	Plausible disease-causing mutations identified on one allele in all or the vast majority of with relevant disorder
**Bialellic**	Plausible disease-causing homozygous or compound heterozygous mutations identified on both alleles in the majority of with relevant disorder
**Both**	Plausible disease-causing mutations identified on either one or both alleles in a relevant disorder where the mono allelic cases cannot be accounted for by false negative screens on the other allele
**Imprinted**	Plausible disease-causing mutations identified on one allele with the parent of origin determining the relevant disorder
**Digenic**	Plausible disease-causing mutations identified on one or both alleles of two different genes causing a relevant disorder where similar mutations of either gene would not
**Hemizygous**	Plausible disease-causing mutations identified on the X chromosome in a male as a cause of a relevant disorder, the disorder being predominantly recessive in female carriers
**X-linked dominant**	Plausible disease-causing mutations identified one copy of the X chromosome in females as a cause of a relevant disorder, includes disorders where heterozygous females and hemizygous males are similarly affected e.g. SMC1A mutations
**Mosaic**	Plausible disease-causing mutations identified on one allele in a proportion of cells with the others being wild-type as a cause of a relevant disorder
**Mitochondrial**	Plausible disease-causing mutations identified on mitochondial DNA where homoplasmy or heteroplasmy are associated with a relevant disorder
**Uncertain**	Plausible disease-causing mutations in which the allele status is not recorded or is unclear with relevant disorder

Supplementary Table 3: G2P Mutational Consequence Categories

Category	Description
**Loss of function**	Where any of the mutations are nonsense, frame-shifting indel, essential splice site mutation, whole gene deletion OR any other mutation where functional analysis demonstrates clear reduction or loss of function
**All missense/in frame**	Where all the mutations described in the data source are either missense or in frame deletions and there is no evidence favoring either loss-of-function, activating or dominant negative effect
**Dominant negative**	Mutation within one allele of a gene that creates a significantly greater deleterious effect on gene product function than a monoallelic loss of function mutation
**Activating**	Mutation, usually missense that results in functional activation of the gene product
**Increased gene dosage**	Copy number variation that increases the functional dosage of the gene
**Cis-regulatory or promotor mutation**	Mutation in cis-regulatory elements that lies outwith the known transcription unit and promotor of the controlled gene
**5' or 3'UTR mutation**	Mutation within the 5' or 3' untranslated region of the transcript which results in mislocalisation or altered stability of RNA molecule
**Uncertain**	Where the exact nature of the mutation is unclear or not recorded

Supplementary Table 4: Total number of variants before and after filtering

	Mean number per sample (+/- stdev) SNP	Mean number per sample (+/- stdev) INDEL
exome-wide GS	90,912 (+/- 3,521)	12,317 (+/- 924)
exome-wide CRC	90,255 (+/- 3,072)	12,214 (+/- 794)
exome-wide DDD	112,693 (+/- 7,480)	14,171 (+/- 1,389)
panel-wide GS	8,449 (+/- 384)	1,210 (+/- 100)
panel-wide CRC	8,407 (+/- 315)	1,209 (+/- 88)
panel-wide DDD	11,169 (+/- 838)	1,498 (+/- 160)
valid G2P GS	2.05 (+/- 1.51)	0.09 (+/- 0.40)
valid G2P CRC	2.21 (+/- 1.83)	0.08 (+/- 0.32)
valid G2P DDD	3.59 (+/- 2.56)	0.19 (+/- 0.50)

Exome-wide SNPs/INDELs: average number of high-quality variants per sample at exome-wide level
Panel-wide SNPs/INDELs: average number of high-quality variants in the genes within the reportable genes in G2PDD gene panel
Valid G2PDD hits: average number per sample of SNPs/INDELs passing all the filtering (i.e. satisfying the variant quality and DDG2P gene allelic requirements); for SNPs - included are all missense variants, regardless of their SIFT/PolyPhen predicted effect

Supplementary Table 5: Number of individuals with variants in Monoallelic LGMDETs surviving filtering using VEP-G2P^{DD} in DDD and GS cohorts

	p-value	OR	95% LCI	95% UCI	# DDD pat	Proportion of reported individuals	average MAF DDD	# GS pat	Proportion of reported individuals	average MAF GS	GS/DDD MAF
splice_donor_variant	0.11	2.3	0.8	6.12	208	0.04	3.72E-06	4	0.03	3.05E-06	0.82
splice_acceptor_variant	0.38	1.9	0.6	5.96	131	0.02	2.04E-06	3	0.02	6.75E-06	3.31
**stop_gained**	**5E-08**	**9**	**2.9**	**28**	**583**	**0.10**	**1.17E-06**	**3**	**0.02**	**4.06E-06**	**3.47**
**frameshift_variant**	**0**	**6.3**	**2.8**	**14.1**	**800**	**0.14**	**1.26E-06**	**6**	**0.04**	**2.56E-06**	**2.03**
start_lost	1				19	0.00	2.72E-06	0	0.00	0	0.00
stop_lost	0.25	0.3	0	2.14	6	0.00	3.01E-06	1	0.01	0	0.00
**inframe_insertion**	**0.03**	**6.1**	**0.9**	**44**	**141**	**0.02**	**4.06E-06**	**1**	**0.01**	**0**	**0.00**
**inframe_deletion**	**0.004**	**3.2**	**1.3**	**7.79**	**361**	**0.06**	**3.97E-06**	**5**	**0.04**	**1.405E-05**	**3.54**
**missense_variant**	**6.5E-07**	**1.8**	**1.4**	**2.26**	**3811**	**0.65**	**4.04E-06**	**118**	**0.84**	**6.77E-06**	**1.68**
coding_sequence_variant	1				18	0.00	3.81E-06	0	0.00	0	0.00
protein_altering_variant	1				5	0.00	0	0	0.00	0	0.00
Total Number of Variants						5875			141		

Monoallelic DDG2P genes, canonical transcripts only . p-value = two-tail Fisher's Exact Test on the number of DDD and GS samples with at least one variant of this type. OR = Odds-ratio on the number of DDD and GS samples with at least one variant of this type (95% LCI and UCI = 95% confidence interval for the OR). Number DDD samples = number of samples in DDD cohort with at least one variant of this type
Proportion of reported variants = proportion of samples having variant of this type out of total reported
Mean MAF DDD = mean ExAC MAF for the DDD variants of this type
missense variants = considering only missense variants for which both SIFT and PolyPhen agree deleterious/damaging
bold text indicates p-value < 0.05 and 95% LCI > 1.0

Supplementary Table 6; Number of individuals with variants in Biallelic LGMDETs surviving filtering using VEP-G2P[DD] in DDD and GS cohorts

	p-value	OR	95% LCI	95% UCI	# DDD pat	Proportion of reported individuals	aver MAF DDD	# GS pat	Proportion of reported individual	aver MAF GS	GS/DDD MAF
splice_donor_variant	0.19	3.9	0.54	28	90	0.03	0.0005448	1	0.01	3E-05	0.05
splice_acceptor_variant	0.26	3.4	0.47	24.6	79	0.03	3.486E-05	1	0.01	2E-05	0.47
**stop_gained**	**0.02**	**4.3**	**1.07**	**17.5**	**198**	**0.06**	**0.0000674**	**2**	**0.03**	**0.0002**	**2.59**
frameshift_variant	0.21	1.6	0.8	3.08	325	0.10	6.208E-05	9	0.13	0.0002	3.64
start_lost	1.00				6	0.00	1.922E-05	0	0.00		0.00
stop_lost	1.00				3	0.00	0.000015	0	0.00		0.00
inframe_insertion	0.40				34	0.01	0.0001447	0	0.00		0.00
inframe_deletion	0.81	0.9	0.38	2.32	110	0.03	0.000185	5	0.07	0.0004	2.32
**missense_variant**	**0.00**	**2.5**	**1.85**	**3.45**	**2297**	**0.73**	**0.0003093**	**48**	**0.72**	**0.0004**	**1.24**
coding_sequence_variant	0.31	0.3	0.04	2.74	8	0.00	3.339E-05	1	0.01	2E-05	0.49
protein_altering_variant	1.00				5	0.00	5.218E-05	0	0.00		0.00
Total Number of Variants					3155			67			

Biallelic DDG2P genes, canonical transcripts only
p-value = two-tail Fisher's Exact Test on the number of DDD and GS samples with at least one variant of this type
OR = Odds-ratio on the number of DDD and GS samples with at least one variant of this type (95% LCI and UCI = 95% confidence interval for the OR)
Number DDD samples = number of samples in DDD cohort with at least one variant of this type
Proportion of reported variants = proportion of samples having variant of this type out of total reported
Mean MAF DDD = mean ExAC MAF for the DDD variants of this type
missense variants = considering only missense variants for which both SIFT and PolyPhen agree deleterious/damaging
bold text indicates p-value < 0.05 and 95% LCI > 1.0

Supplementary Table 7: Number of individuals with variants in Monoallelic LGMDETs surviving filtering using VEP-G2P^{DD} in CRC and GS cohorts

	**p-value**	**OR**	**95% LCI**	**95% UCI**	**# CRC pat**	**Prop^{n} reported individual**	**aver MAF CRC**	**# GS pat**	**Prop^{n} reported individual**	**average MAF GS**	GS/ CRC MAF
splice_donor_variant	1.00	1.22	0.37	4.09	8	0.03	8.8E-06	4	0.03	3.1E-06	0.35
splice_acceptor_variant	0.68	0.61	0.12	3.03	3	0.01	7.4E-06	3	0.02	6.8E-06	0.92
stop_gained	0.55	1.63	0.43	6.21	8	0.03	2.6E-06	3	0.02	4.1E-06	1.57
frameshift_variant	0.21	1.86	0.73	4.73	18	0.07	2.1E-06	6	0.04	2.6E-06	1.25
start_lost	0.29				3	0.01	2.8E-06	0	0.00		0.00
stop_lost	1.00	0.61	0.04	9.76	1	0.00	0	1	0.01	0	
inframe_insertion	0.66	2.45	0.27	22	4	0.02	2E-06	1	0.01	0	0.00
inframe_deletion	0.35	1.73	0.62	4.84	14	0.05	9.1E-06	5	0.04	1.4E-05	1.54
missense_variant	0.61	1.09	0.82	1.45	204	0.77	6.9E-06	118	0.84	6.8E-06	0.98
coding_sequence_variant	1.00				1	0.00	4.2E-06	0	0.00		0.00
protein_altering_variant					0	0.00		0	0.00		
Total Number Variants						264			141		

Monoallelic DDG2P genes, canonical transcripts only
p-value = two-tail Fisher's Exact Test on the number of CRC and GS samples with at least one variant of this type
OR = Odds-ratio on the number of CRC and GS samples with at least one variant of this type (95% LCI and UCI = 95% confidence interval for the OR)
Number CRC samples = number of samples in CRC cohort with at least one variant of this type
Proportion of reported variants = proportion of samples having variant of this type out of total reported
Mean MAF CRC = mean ExAC MAF for the CRC variants of this type
missense variants = considering only missense variants for which both SIFT and PolyPhen agree deleterious/damaging
bold text indicates p-value < 0.05 and 95% LCI > 1.0

Supplementary Table 8: Number of individuals with variants in LGMDETs surviving filtering using VEP-G2P^{DD} in CRC and GS cohorts

	p-value	OR	95% LCI	95% UCI	# CRC pat	Proportion of reported individuals	average MAF CRC	# GS pat	Proportion of reported individuals	average MAF GS	GS/CRC MAF
splice_donor_variant	1.00	1	0.11	13.5	2	0.02	2E-05	1	0.01	3E-05	1.68
splice_acceptor_variant	1.00	2	0.19	17.7	3	0.03	4E-05	1	0.01	2E-05	0.40
stop_gained	0.50	2	0.44	10.41	7	0.06	0.0002	2	0.03	0.0002	0.77
frameshift_variant	0.33	1	0.24	1.53	9	0.08	0.0003	9	0.13	0.0002	0.74
start_lost					0	0.00	0	0	0.00	0	
stop_lost	0.53				2	0.02	0	0	0.00	0	
inframe_insertion	1.00				1	0.01	4E-06	0	0.00	0	0.00
inframe_deletion	0.31	0	0.13	1.81	4	0.03	0.0003	5	0.07	0.0004	1.55
missense_variant	0.56	1	0.78	1.67	88	0.75	0.0005	48	0.72	0.0004	0.83
coding_sequence_variant	0.38				0	0.00	0	1	0.01	2E-05	
protein_altering_variant	1.00				1	0.01	0	0	0.00	0	
Total Number of Variants					117			67			

Biallelic DDG2P genes, canonical transcripts only
p-value = two-tail Fisher's Exact Test on the number of CRC and GS samples with at least one variant of this type
OR = Odds-ratio on the number of CRC and GS samples with at least one variant of this type (95% LCI and UCI = 95% confidence interval for the OR)
Number CRC samples = number of samples in CRC cohort with at least one variant of this type
Proportion of reported variants = proportion of samples having variant of this type out of total reported
Mean MAF CRC = mean ExAC MAF for the CRC variants of this type
missense variants = considering only missense variants for which both SIFT and PolyPhen agree deleterious/damaging

Supplementary Table 9: Number of individuals with variants in Monoallelic LGMDETs surviving filtering using VEP-G2P^{Cancer} in CRC and GS cohorts

	p-value	OR	95% LCI	95% UCI	# CRC pat	Proportion of reported individuals	average MAF CRC	# GS pat	Proportion of reported individuals	average MAF GS	GS/CRC MAF
splice_donor_variant	0.66	2.5	0.27	22	4	0.07	3E-06	1	0.04	4E-06	1.33
splice_acceptor_variant	1.00	0.6	0.04	9.76	1	0.02	0	1	0.04	9E-06	
stop_gained	0.72	1.8	0.37	9.16	6	0.10	2E-06	2	0.08	5E-06	2.67
frameshift_variant	1.00	0.8	0.18	3.65	4	0.07	2E-06	3	0.12	1E-06	0.67
start_lost	1.00				1	0.02	4E-06	0	0.00	0	0.00
stop_lost					0	0.00	0	0	0.00	0	
inframe_insertion	1.00				1	0.02	0	0	0.00	0	
inframe_deletion	0.53				2	0.03	4E-06	0	0.00	0	0.00
missense_variant	0.40	1.3	0.74	2.3	40	0.68	8E-06	19	0.73	6E-06	0.69
coding_sequence_variant					0	0.00	0	0	0.00	0	
protein_altering_variant					0	0.00	0	0	0.00	0	
Total Number of Variants					59			26			

Monoallelic CancerG2P genes, canonical transcripts only
p-value = two-tail Fisher's Exact Test on the number of CRC and GS samples with at least one variant of this type
OR = Odds-ratio on the number of CRC and GS samples with at least one variant of this type (95% LCI and UCI = 95% confidence interval for the OR)
Number CRC samples = number of samples in CRC cohort with at least one variant of this type
Proportion of reported variants = proportion of samples having variant of this type out of total reported
Mean MAF CRC = mean ExAC MAF for the CRC variants of this type
missense variants = considering only missense variants for which both SIFT and PolyPhen agree deleterious/damaging

Supplementary Table 10: Number of individuals with variants in biallelic LGMDETs surviving filtering using VEP-G2P^{Cancer} in CRC and GS cohorts

	p-value	OR	95% LCI	95% UCI	# CRC pat	average MAF CRC	# GS pat	average MAF GS
splice_donor_variant					0		0	
splice_acceptor_variant					0		0	
stop_gained					0		0	
frameshift_variant	0.29				3	5E-06	0	
start_lost								
stop_lost								
inframe_insertion								
inframe_deletion	1.00				1	0	0	
missense_variant	0.16				5	0.0002	0	
coding_sequence_variant					0		0	
protein_altering_variant					0		0	
Total number of Variants					9		0	

Biallelic CancerG2P genes, canonical transcripts only
p-value = two-tail Fisher's Exact Test on the number of CRC and GS samples with at least one variant of this type
OR = Odds-ratio on the number of CRC and GS samples with at least one variant of this type (95% LCI and UCI = 95% confidence interval for the OR)
Number CRC samples = number of samples in CRC cohort with at least one variant of this type
Proportion of reported variants = proportion of samples having variant of this type out of total reported
Mean MAF CRC = mean ExAC MAF for the CRC variants of this type
missense variants = considering only missense variants for which both SIFT and PolyPhen agree deleterious/damaging

Supplementary Methods

G2P web application as a curation tool

The G2P web application (https://www.ebi.ac.uk/gene2phenotype/) is a panel development and curation tool. Curators are assigned access to one or more panels and can log into the website in order to edit a dataset. New entries are initiated by selection of a gene symbol from the list of preloaded genes with their associated Ensembl identifiers and HGNC symbols (these are updated after each new Ensembl release). Searching with previously used gene symbols is supported, but results are shown using the current gene symbol. The search results page lists all LGMDET that are already stored in the database for the given gene. If the LGMDET is not already in the database for the panel of interest, the curator follows the link that guides them through the creation of a new LGMDET. During the process, the curator specifies which panel(s) the new pair/thread belongs to and the associated disease name. After completing the initial creation process the curator is directed to the new LGMDET page. The layout of the LGMDET page is the same regardless of the login status, but curators are presented with additional editing options. The details of edits changing the LGMDET confidence and the disease mechanism is stored. This history lists the creation date, name of the curator, new value and the edit action (create or edit) and is only visible to curators who are logged in and have sufficient edit rights for the respective panel.

G2P implementation details

The G2P web application is built with the Perl Mojolicious web framework. We use the Bootstrap framework and its HTML and CSS-based design templates; we use jQuery, a JavaScript library, for the front-end development. Our data are stored in a MySQL relational database. The ensembl-gene2phenotype API provides access to the database and supports data retrieval and data edits. The database schema and API have been developed according to the design principles of existing Ensembl databases and APIs. The ensembl-gene2phenotype API inherits all methods for manipulating data in the underlying database from the Ensembl core API[1].

G2P VEP plugin logic

The VEP-G2P plugin (https://www.ebi.ac.uk/gene2phenotype/g2p_vep_plugin) identifies possible disease-causing variants (i.e. "valid hits") by applying a set of filtering rules. If a variant passes all filtering rules it will be further considered to decide if a sufficient number of variants that passed the filtering overlap a transcript and fulfil the allelic requirement of the transcript's gene. The sufficient

number of variants is determined by the allelic requirement of the gene: for a biallelic gene, at least 2 heterozygous variants which pass all filtering rules and are located in the same transcript are required, or 1 homozygous variant passing all filtering rules; for a monoallelic gene, either 1 heterozygous variant or 1 homozygous variant passing all filtering rules is required.

The filtering rules that are applied to each input variant are: **1.** The variant overlaps a G2P gene; **2.** The predicted functional consequence is considered severe. The default list of severe consequences contains the following terms: splice_donor_variant, splice_acceptor_variant, stop_gained, frameshift_variant,stop_lost,initiator_codon_variant, inframe_insertion, inframe_deletion, missense_variant, coding_sequence_variant, start_lost, transcript_ablation, transcript_amplification, protein_altering_variant; **3.** If the variant allele has been observed in a reference population (ie. from 1000 Genomes Project, ESP, gnomAD, UK10K or TOPMed), the observed allele frequencies must be below a given threshold. The default threshold for observed allele frequency for a variant in a bi-allelic gene is 0.005 and for a variant in a mono-allelic gene is 0.0001. The default parameters for both frequency and consequence can be altered by users.

If the input variant passes all filtering rules, the overlapping transcript is used to retrieve the most recent HGNC gene symbol and all previously assigned symbols for the transcript's gene. The list of gene symbols is used to look up data from the input file. It is important to note that G2P reports back all the gene's transcripts with a valid hit with the canonical transcript annotated accordingly.

VEP-G2P plugin input data format

The plugin requires as input a file which lists genes of interest and their allelic requirements. Files for the G2P panels can be downloaded for individual panels from the G2P website https://www.ebi.ac.uk/gene2phenotype/downloads. Such input files can be generated for any gene set. The file needs to be tab-delimited and must contain at least two columns; the first listing the gene symbol and the second listing the allelic requirement. The customized file must start with a header line. Recognized header fields are 'gene symbol' and 'allelic requirement'. Each row lists a pair of gene symbol and allelic requirement. If the gene has more than one allelic requirement a row for each allelic requirement can be provided or allelic requirements can also be separated by a semi-colon. The plugin also accepts PanelApp data files (https://panelapp.genomicsengland.co.uk). A PanelApp data file is recognized by its header fields: 'Gene_Symbol' and 'Model_Of_Inheritance' which are the equivalents

of ‘gene symbol’ and ‘allelic requirement’. Variant data can be input in VCF or a simple tab delimited format.

Computational time optimization

In order to reduce the VEP-G2P computational time, from the WES VCFs for each cohort we extracted only variants from the genomic regions containing the genes in the $G2P^{DD}$ (~145Mbp) and $G2P^{Cancer}$ (~8Mbp) gene panels. This results in ~10-fold decrease in the number of variants to be analysed for the $G2P^{DD}$ panel and ~150-fold decrease for the $G2P^{Cancer}$ panel. Computational time on genomic variant sets can also be substantially reduced using the '--transcript_filter' option to select specific genes or transcripts for analysis.

The VEP-G2P time taken for GS cohort (315 individuals) was 13 minutes for the $G2P^{Cancer}$ panel (3607 variant sites) and 212 minutes for the $G2P^{DD}$ panel (53130 variant sites); CRC cohort (517 individuals) took 29 minutes for the $G2P^{Cancer}$ panel (4490 variant sites) and 336 minutes for the $G2P^{DD}$ panel (64961 variant sites). The 7357 DDD individuals were split in 10 batches of ~736 samples and ~134000 variants in each on average, and their simultaneous analysis for the $G2P^{DD}$ panel took between 533 and 717 minutes. It should be noted that there is non-trivial time overhead required for running the VEP-G2P plugin as compared to the VEP alone. gnomAD data for GRCh37 will be added for Ensembl release/95 due in December 2018 and this should reduce run time.

Gene sets for supported assemblies

The default gene set for VEP variant annotation is the Ensembl/GENCODE gene set. The Ensembl/GENCODE gene set is the full merge of Ensembl evidence-based transcript predictions with Ensembl manual annotation[2]. VEP supports annotation for human GRCh37 and GRCh38 assemblies. Ensembl/GENCODE data on GRCh37 is provided as a stable archive since the release of the GRCh38 assembly. The gene annotation on the GRCh37 archive is based on Ensembl/GENCODE data from release 75 (Ensembl release/90: GRCh37.p13 February 2014). The gene set for GRCh38 is updated twice per year. The RefSeq gene models can also be used in VEP annotation.

Allele frequency annotation

VEP can use local cache files which are built from the variants stored in an Ensembl variation database together with allele frequencies from the 1000 Genomes Project, the NHLBI Exome Sequencing Project and the gnomAD exome data to provide allele frequency data. The variation data

for GRCh37 are updated roughly annually whereas the database for GRCh38 is updated four times per year. The cache files contain allele frequencies for variants which have been assigned RefSNP (rsID) identifiers. The VEP-G2P plugin uses the allele frequencies from the cache files for variant filtering but can also add allele frequencies from other sequencing projects like TOPMed, UK10K and the gnomAD whole genome data. The plugin uses a function implemented in the Ensembl API to match input variants directly to alleles in VCF files, resolving different normalisation approaches that may have been used. VEP can also deal with gnomAD's representation of multi-allelic variants on one VCF line. In order to derive the correct co-located variant allele for an input allele VEP normalizes the input variant allele and the co-located allele independently to arrive at a potential match. The algorithm is outlined on the VEP documentation pages [http://www.ensembl.org/info/docs/tools/vep/script/vep_other.html#colocated]. Searching additional allele frequency resources slows down the overall VEP run but provides more extensive frequency annotation.

References:

1 Ruffier, M. *et al.* Ensembl core software resources: storage and programmatic access for DNA sequence and genome annotation. *Database (Oxford)* **2017** (2017).

2 Harrow, J. *et al.* GENCODE: the reference human genome annotation for The ENCODE Project. *Genome Res* **22**, 1760-1774 (2012).
