# Supplementary material for: Flexible and scalable diagnostic filtering of genomic variants using G2P with Ensembl VEP
Source: Nat Commun. 2019 May 30;10:2373. doi: 10.1038/s41467-019-10016-3 (PMC6542828; doi:10.1038/s41467-019-10016-3)
Supplement: Supplementary file 3 — Description of Additional Supplementary Files [file 41467_2019_10016_MOESM3_ESM.pdf]

Description File for Additional Supplementary Data

File Name: Supplementary Data 1
Description: Summary of January 2018 Freeze of G2PDD dataset

File Name: Supplementary Data 2
Description: Summary of January 2018 Freeze of G2PCancer dataset

File Name: Supplementary Data 3
Description: Per gene assessment of background noise in G2P-DD

File Name: Supplementary Data 4
Description: Per gene assessment of background noise in G2P-Cancer
